# Assessment of a 2-Step Urine Culture Ordering Process for Detecting Asymptomatic Bacteruria Among Hospitalized Patients

**DOI:** 10.1001/jamanetworkopen.2019.21665

**Published:** 2020-02-21

**Authors:** Maureen T. Taylor, Janine McCready, Thomas Havey, Sahejpreet Kaur, Jeff Powis

**Affiliations:** 1 Division of Infectious Diseases, Department of Medicine, Michael Garron Hospital, Toronto, Ontario, Canada; 2 William Osler Health System, Toronto, Ontario, Canada; 3 Royal College of Surgeons in Ireland, Dublin, Ireland

## Abstract

This quality improvement study evaluates the association of a 2-step urine culture ordering process with the number of urine cultures processed each month and the detection of asymptomatic bacteruria and candiduria among patients hospitalized in an acute care setting.

## Introduction

Educational interventions attempting to reduce the treatment of asymptomatic bacteruria and candiduria (ASB/C) have been largely unsuccessful. Modifying how urine cultures (UCs) are ordered, processed, and reported may be a more effective strategy for reducing the overdiagnosis of ASB/C.^1^ A 2-step UC ordering process was associated with a reduction in the number of UCs and antimicrobial treatments of ASB/C in the emergency department,^2^ and we evaluated a similar intervention among patients hospitalized in an acute care setting.

## Methods

This quality improvement study was approved by the Michael Garron Hospital research ethics board. Because the project was deemed a quality improvement evaluation with minimal risk, patient consent was waived. This report was structured according to the Standards for Quality Improvement Reporting Excellence (SQUIRE) reporting guideline for quality improvement studies.^3^

A time series analysis was conducted to evaluate the association of a 2-step UC ordering process with the number of processed UCs per 100 patient-days (primary outcome) and antimicrobial use, intensive care unit transfers, critical care outreach assessment, in-hospital mortality, length of stay, and 7-day readmission rates (secondary outcomes) at an urban community hospital with 515 beds. Before the intervention, UCs could be submitted by nurses, according to medical directives, or by physicians, based on clinical suspicion of urinary tract infection, and all UCs were processed by the microbiology laboratory. The 2-step process involved collection of UCs by nurses based on clinical symptoms. The sample was stored in a container with preservatives, allowing it to be held for 48 hours in the laboratory until a separate physician order triggered processing. Urine cultures were not processed unless a separate physician order was received. Urinalysis was not required at the time of UC but could be ordered at the discretion of the physician. Education was provided to nurses and physicians regarding the ordering process, but no specific education was provided regarding indications for urine culturing. All catheter and noncatheter urine specimens collected from May 2015 to April 2016 (ie, the preintervention period) and May 2016 to April 2017 (ie, the postintervention period) were eligible for analysis. A random medical record audit was conducted with records from 100 patients in both the preintervention and postintervention periods to determine ordering clinician, indication for ordering the UC, and antimicrobial use. The postintervention population was stratified to ensure proportions of processed UCs were the same as the entire postintervention population.

Analyses were conducted via linear regression and a χ^2^ test of independence using R version 3.0.2 (R Project for Statistical Computing) with *P* < .05 considered significant. All tests were 2-tailed.

## Results

A total of 1344 ordered UCs were included in the preintervention analysis, and 866 ordered UCs were included in the postintervention analysis. Preintervention and postintervention populations were comparable (UCs from patients aged 18-65 years: 327 [32.1%] vs 224 [30.8%]; women: 561 [55.0%] vs 387 [53.2%]; patients located in medical units: 316 [31.0%] vs 241 [33.1%]); however, there were more pediatric cases in the postintervention period than the preintervention period (56 [7.7%] vs 49 [4.8%]; *P* = .02) (Table). There was no change in secondary outcomes (Table). Comparing the preintervention period with the postintervention period, the intervention was associated with a significant reduction in processed UCs per 100 patient-days (2.29 vs 1.17 per 100 patient-days; *P* = .001) (Figure). In the first month of the intervention, the modeled rate was 1.03 (95% CI, 0.85 to 1.21). The amount of change associated with the intervention (adjusted for the estimated temporal trend) was −0.03 per month (95% CI, −0.10 to −0.05; *P* = .02). This represents a 0.93 absolute reduction (95% CI, 0.59 to 1.27; *P* < .001). Compared with the preintervention period, the postintervention period saw a reduction in monthly processed UCs in all clinical areas except the pediatric unit (UCs per 100 patient-days from medical wards: 1.92 vs 0.78; *P* < .001; surgical wards: 2.11 vs 0.96; *P* < .001; intensive care unit: 3.11 vs 1.64; *P* < .005; pediatric ward: 3.08 vs 4.06; *P* = .68). In the preintervention period, nurses ordered 45 of 100 UCs (45.0%), based on medical directive, of which 32 (71.1%) had no indication based on accepted guidelines.^4,5^ Similar proportions of patients in the preintervention and postintervention periods had empirical antimicrobials initiated (38 of 100 [38.0%] vs 37 of 100 [37.0%]), mostly for nonurinary indications (28 of 38 [73.7%] vs 30 of 37 [81.1%]). Among patients not receiving empirical antimicrobials, the intervention was associated with a significant decrease in the detection of ASB/C (18 of 62 [29.0%] in the preintervention period; 7 of 63 [11.1%] in the postintervention period; *P* = .01). Treatment of ASB/C was infrequent (3 of 18 episodes [16.7%] in the preintervention period; 1 of 7 episodes [14.3%] in the postintervention period) and did not change significantly (*P* = .90).

**Table.** Patient Characteristics and Outcomes

Characteristic	No. (%)		*P* Value
	Preintervention Period	Postintervention Period	
UCs ordered, No.^a^	1344	866	NA
Unique patients, No.	1020	728	NA
Age distribution, y			
<18	46 (4.5)	53 (7.3)	.02
18-65	327 (32.1)	224 (30.8)	.60
>65	647 (63.4)	451 (62.0)	.56
Sex			
Women	561 (55.0)	387 (53.2)	.48
Men	459 (45.0)	341 (46.8)	
Unit location			
Medical	316 (31.0)	241 (33.1)	.38
Surgical	243 (23.8)	180 (24.7)	.71
Pediatrics	49 (4.8)	56 (7.7)	.02
Critical care	80 (7.8)	65 (8.9)	.47
UTI diagnosis at admission			
Fever, unspecified	5 (0.5)	2 (0.3)	.71
Lower UTI	16 (1.6)	10 (1.3)	.84
Other or unspecified sepsis	26 (2.6)	19 (2.6)	>.99
Upper UTI	10 (1.0)	4 (0.60	.42
HIG score, mean (SD)	4.0 (8.1)	3.5 (5.5)	.25
Outcomes, mean (SD), No.			
Monthly UCs processed per 100 patient-days	2.3 (0.3)	1.2 (0.2)	<.001
Antimicrobial DOT per 100 patient-days, d	20.8 (6.5)	21.7 (7.2)	.89
LOS, d	16.6 (24.3)	16.0 (20.0)	.25
7-d readmission rates per 100 patient-days	7.9 (2.6)	8.5 (3.0)	.55
In-hospital mortality rates per 100 patient-days	10.7 (3.7)	12.1 (3.8)	.40
ICU transfer per 100 patient-days	2.8 (0.5)	2.4 (0.9)	.11
Critical care outreach assessment per 100 patient-days	9.9 (3.2)	8.7 (2.9)	.52

Abbreviations: DOT, days of therapy; HIG, health-based allocation model inpatient group; ICU, intensive care unit; LOS, length of stay; NA, not applicable; UC, urine culture; UTI, urinary tract infection.

^a^ Number of UCs ordered includes duplicates.

**Figure.**
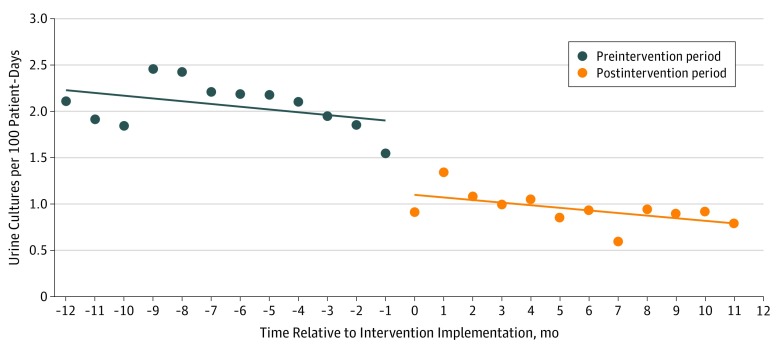
Monthly Inpatient Urine Cultures Processed per 100 Patient-Days Before and After the Implementation of a 2-Step Urine Culture Ordering Process Lines represent linear regression lines of best fit, and dots represent the monthly UCs per 100 patient-days for each month of study.

## Discussion

Before the intervention, patients who were hospitalized often had UCs completed by nursing staff for nonspecific symptoms. In this quality improvement study, a 2-step UC ordering process was associated with a nearly 50% reduction in the number of UCs processed and a reduction in the detection of ASB/C. Our intervention avoided unnecessary UCs while maintaining nurses’ autonomy to collect them. Not culturing urine maximizes cost avoidance compared with analytical or postanalytical interventions. The advantage of our 2-step ordering process compared with a reflex policy based on pyuria is that the decision to process a UC in our intervention was based on clinical assessment by a physician rather than the result of urinalysis alone. Asymptomatic pyuria can occur in as many as 90% of elderly patients, for whom processing UCs is likely to yield a diagnosis of ASB/C and unnecessary antimicrobial therapy.

There was no significant change in overall antimicrobial use or treatment of ASB/C. We attribute this to the fact that most empirical antimicrobials were provided for nonurinary indications and that the treatment of ASB/C was far lower than expected from the literature because of our antimicrobial stewardship program.^6^

Our study has several limitations. First, it was conducted at a single community hospital, with a medical directive for nurses to order UCs. Therefore, it may not apply to all acute care settings. Additionally, we conducted a medical record audit on a random sample of patients who, by chance, may not be representative of the larger sample. Although pediatric cases made up a small proportion of our study population, the 2-step UC ordering process was not associated with reductions in the number of UCs processed in this population, and as a result, our intervention would be anticipated to have a smaller association with reductions in the number of processed UCs if the population under evaluation included a larger proportion of pediatric patients. Furthermore, a computer-order entry system is required to help physicians order UCs after clinical assessment.

This 2-step UC ordering process for patients in an acute care setting was associated with a reduction in the number of UCs processed as well as a reduction in the detection of ASB/C. Our intervention was not associated with the elimination of the detection of ASB/C and, therefore, should be considered part of broader strategy to prevent antimicrobial use for ASB/C. Further evaluation would be necessary to determine whether the intervention was associated with reductions in antimicrobial treatment of ASB/C in an acute care setting where ASB/C is treated at usual rates.
